# Matricellular Proteins in Bladder Cancer: Context-Dependent Roles in Tumor Promotion and Suppression

**DOI:** 10.3390/ijms27156807

**Published:** 2026-07-29

**Authors:** Azamat Akhmetkaliyev, José Héctor Gibrán Fritz García, Eva Sonnenberg-Riethmacher, Dieter Riethmacher

**Affiliations:** 1 Department of Biomedical Sciences, School of Medicine, Nazarbayev University, Astana 010000, Kazakhstan; 2 Faculty of Biology, Johannes Gutenberg University Mainz, 55128 Mainz, Germany; 3 Human Development and Health, Faculty of Medicine, University of Southampton, Southampton SO16 6YD, UK

**Keywords:** bladder cancer, urothelial carcinoma, extracellular matrix, tumor microenvironment, matricellular proteins, tenascin-C, osteopontin, SPARC, periostin, CYR61

## Abstract

Bladder cancer (BLCA) is a common and heterogeneous malignancy in which disease progression is driven not only by tumor-intrinsic alterations but also by dynamic interactions within the tumor microenvironment (TME). Increasing evidence positions the extracellular matrix (ECM) as a critical regulator of these processes. Matricellular proteins (MCPs), a group of nonstructural ECM-associated molecules, have emerged as key modulators of tumor–stroma communication. In BLCA, MCPs have been reported to display divergent, and in some cases opposing, associations or functions, with the same protein participating in both tumor promotion and suppression. Here, we review current evidence on the function of MCPs in BLCA and synthesize their bidirectional roles in carcinogenesis. MCPs contribute to tumor progression by promoting invasion, epithelial–mesenchymal transition (EMT), angiogenesis, and metastatic niche formation. At the same time, MCPs can restrain tumor growth by inhibiting angiogenesis, stabilizing ECM organization, inducing cell cycle arrest, and maintaining epithelial integrity. A key concept emerging from this body of evidence is the context-dependent functional plasticity of MCPs. We propose that MCP-associated phenotypes in BLCA may be influenced by contextual factors, including isoform diversity arising from alternative splicing and post-translational modifications, spatial compartmentalization within tumor and stromal niches, tumor microenvironmental composition, and molecular subtype. However, the level of supporting evidence differs substantially among MCPs, and direct BLCA-specific mechanistic evidence remains limited for many proposed relationships. These factors, therefore, provide a framework for interpreting divergent findings rather than representing universally established determinants of MCP function. Recognizing MCPs as context-sensitive regulators rather than fixed tumor-promoting or tumor-suppressing entities provides a unifying framework for understanding their roles in BLCA. This could be an important step for therapeutic targeting, encouraging effective strategies to consider and incorporate the molecular and microenvironmental context in which MCPs operate.

## 1. Introduction

BLCA is the most prevalent malignancy affecting the urinary tract [1]. In 2022, this cancer was the 9th most commonly diagnosed (613,791 new cases), and the 13th leading cause of cancer-related mortality (220,349 deaths) worldwide [2]. The burden of BLCA differs substantially across geographic regions, with Southern and Western Europe showing the highest number of recorded cases, whereas Northern Africa has the highest mortality rates. The disease predominantly affects older adults and men, with an incidence rate almost four times higher in men than in women. It is estimated that by 2040, the annual numbers of newly diagnosed BLCA cases and BLCA-associated deaths will rise by 72.8% and 86.6%, respectively, compared with those in 2020 [3].

BLCA originates from the urothelium, a specialized epithelium lining the inner surface of the bladder [4], and is clinically classified into two main forms: non-muscle-invasive (NMIBC) and muscle-invasive (MIBC) [5]. NMIBC accounts for the vast majority of diagnosed cases and is associated with high recurrence rates, yet a fairly favorable survival prognosis [6]. In contrast, MIBC possesses a high metastatic potential, considerably worsening clinical outcomes [7]. Despite significant advances in early diagnosis, risk stratification, and the development of novel therapies, the molecular mechanisms driving recurrence, progression, and, particularly, the transition from superficial to invasive malignancy remain poorly understood.

Increasing evidence suggests that the mechanisms underlying BLCA cannot be solely explained by alterations in genetic and epigenetic programs. Instead, it is currently viewed as a complex interplay between the urothelial cells and the TME, including stromal, vascular, and immune compartments together with the ECM [8,9,10]. In this context, the ECM is now understood to actively shape tumor behavior rather than merely serve as structural support [11,12].

The ECM is a highly dynamic signaling platform integrating biochemical ligands, mechanical signals [13], proteolytic remodeling, and multicellular communication within the TME [14]. Changes in the ECM influence cell signaling, cellular adhesion dynamics, migratory properties, angiogenesis, and immune–stromal interplay [15]. Within this platform, the distinction between structural and regulatory ECM components becomes particularly important. While structural proteins such as collagen, laminin, and fibronectin serve as scaffolds maintaining tissue architecture, MCPs serve as nonstructural extracellular regulators of tumor–stroma communication [16,17]. In particular, MCPs modulate how cells interpret and respond to tumor environments, affecting tumor–stroma communication and influencing proliferation, invasion, epithelial–mesenchymal plasticity, and immune remodeling [18]. Importantly, the functional identity of MCPs is context-dependent [18,19,20] and may vary according to their source, subcellular and tissue localization, molecular isoform, and changes in the tumor environment itself. As a result, the same MCP can either facilitate or restrain cancer progression in a given biological context. In this review, we first examine individual MCPs according to their pro-tumorigenic and anti-tumorigenic roles in BLCA and then suggest putative determinants that may drive context-dependent switching between these opposing functions.

## 2. The ECM in BLCA Evolution: From Structural Scaffold to Signaling Platform

### 2.1. Bladder Wall Architecture and ECM Remodeling During BLCA Progression

The normal bladder wall has a clearly defined, hierarchically organized layered structure consisting of the urothelium, lamina propria, muscularis propria, and the serosal layer [21,22]. The urothelium lies on the basement membrane, and its ECM is composed mostly of collagen type IV, various laminin isoforms, nidogen, fibronectin, and heparan sulfate proteoglycan [23,24]. Together, they support epithelial polarity [25], establish the equilibrium of proliferative capacity [26], and serve as a physical barrier that restricts cell migration [27]. The lamina propria is the next structure beneath the urothelium. It forms vascularized connective compartments containing immune cells, fibroblasts that produce collagen types I and III, fibronectin, and neural elements [28]. The following layer is the muscularis propria, composed of smooth muscle bundles [29]. Under physiological conditions, an equilibrium between matrix synthesis and proteolytic turnover is fundamental for maintaining the integrity of local architecture, but this homeostasis is disturbed during carcinogenesis [30].

ECM remodeling has gained attention as an early event in BLCA pathogenesis that precedes disease onset. A transcriptomic study identified that ECM-related gene profiles are enriched in tumor-associated urothelium and carcinoma in situ (CIS) before invasive commitment, along with a defined specific molecular subtype associated with progression [31]. Consistently, collagen topography begins to change during inflammation, and these changes progress with bladder tumor evolution [32], indicating that ECM changes can accompany and likely contribute to disease evolution. With BLCA development, the ECM shifts from a structural unit to an active signaling network [33]. Stromal fibroblasts, initially providing inhibitory and differentiation signals such as transforming growth factor β1 (TGF-β1) and bone morphogenetic proteins (BMPs), acquire characteristics of cancer-associated fibroblasts (CAFs). These activated cells deposit collagens, produce tumor growth factors, matrix-remodeling enzymes, and other factors that promote matrix reorganization, cell migration, and immune evasion [10].

As lesions evolve, the basement membrane becomes impaired, weakening the epithelial polarity and facilitating invasion into the underlying stroma and muscle [10]. Collagen-containing matrices actively signal via integrins and discoidin domain receptors [34,35], whereas proteolysis-mediated remodeling increases the availability of matrix-bound growth factors and pro-invasive molecules [34]. Simultaneously, collagen crosslinking increases matrix stiffness, which in turn results in activation of integrins, focal adhesion, and YAP/TAZ signaling, further contributing to tumor progression [36,37]. Matrix stiffness increases with BLCA progression, promoting stem-like tumor features via the WNT/β-catenin signaling pathway and linking ECM mechanisms to aggressive tumor phenotypes [38]. The relevance of stromal remodeling is reinforced by clinical data associating fibroblast activation protein-α (FAP) expression with a high risk of transition from NMIBC to MIBC [39], further underscoring the functional importance of stromal activation even before the muscle invasion. Importantly, these ECM-associated changes are more appropriately viewed through the framework of epithelial–mesenchymal plasticity (EMP) rather than a binary EMT. In this model, malignant cells can show dynamic intermediate or hybrid epithelial/mesenchymal states, retaining epithelial features while acquiring mesenchymal properties [40,41]. These states may be reversible and are shaped by extracellular cues including matrix stiffness, integrin signaling, growth factors, inflammatory mediators, and interactions with stromal and immune cells [42,43]. Thus, in BLCA, ECM remodeling and MCP-associated signaling may not necessarily drive complete mesenchymal conversion, and instead, they may stabilize, induce, or enable transitions between plastic cell states that support invasion, dissemination, therapy resistance, and subsequent colonization at metastatic sites. Taken together, these observations suggest that BLCA progression should not be considered simply as tumor cell penetration through the basement membrane, but as a coordinated economical transition involving basement-membrane failure, stromal activation, collagen reorganization, matrix stiffening, and dynamic epithelial–mesenchymal plasticity. In this dynamic environment, regulatory ECM-associated proteins that organize extracellular signaling become particularly relevant, providing the conceptual basis for examining MCPs in the following section.

### 2.2. Matricellular Proteins: Nonstructural Regulators of ECM Signaling

MCPs encompass an array of nonstructural ECM-associated proteins that serve as primary regulators of cell-to-matrix communication rather than just providing structural support for tissue architecture. This large family includes several diverse protein groups: tenascin, thrombospondin (TSP), secreted protein acidic and rich in cysteine (SPARC), cellular communication network (CCN), small leucine-rich proteoglycan families (SLRP), osteopontin, and periostin [19,44,45,46,47].

**Figure 1:**
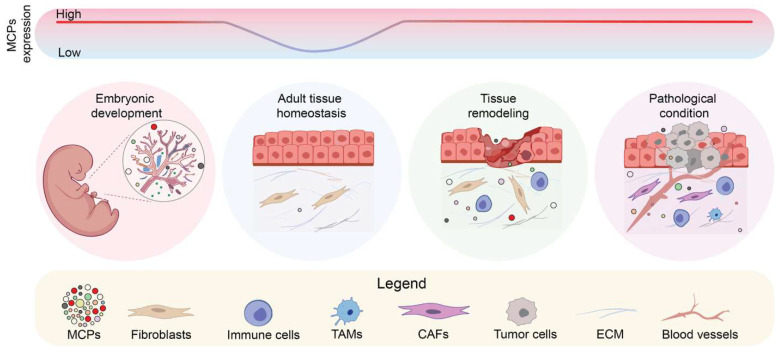
Overview of MCPs expression patterns during development and disease. The expression of MCPs is high during development, tissue remodeling (injury, wound healing, fibrosis), and pathological conditions (chronic inflammation and cancer), while low expression is observed in homeostatic adult tissue.

Despite structural heterogeneity, MCPs share a common regulatory role in tissue remodeling and intercellular signaling. Generally, expression of MCPs is low under homeostatic conditions in adult tissues, but they are robustly expressed during tissue remodeling (i.e., embryonic development, wound healing, fibrosis, chronic inflammation, and cancer) [48,49,50,51] (Figure 1). They can establish multivalent interactions within the TME with cell surface receptors (particularly integrins and proteoglycans) [19], soluble mediators (growth factors, cytokines, proteases) [45], and structural ECM components (collagens) [52], thereby serving as important extracellular signaling organizers. Moreover, MCPs can orchestrate signaling flow by amplifying, dampening, or redirecting it depending on the biological context [18,53].

Another defining characteristic of MCPs is their functional pleiotropy. A single protein can assume distinct functions depending on its spatial and temporal context, a phenomenon referred to as moonlighting [54]. Accordingly, in cancer, a given protein may exert either pro-tumorigenic or anti-tumorigenic effects depending on the cellular, molecular, and microenvironmental conditions in which it operates. This complex multifunctional output allows MCPs to affect multiple levels of cancer progression, affecting cell survival, migratory directionality, proteolytic activity, inflammatory tone, and vascular responsiveness [18,55] (Table 1). This is especially relevant for BLCA, where spatial distribution has already been shown to impact prognostic outcomes for patients [56]. Therefore, the apparent dual role of MCPs should not be viewed as a discrepancy but rather as a reflection of the functional adaptability of ECM signaling networks during cancer evolution.

## 3. Matricellular Proteins Involved in Tumor-Promoting Programs

A growing body of evidence links members of multiple MCP families with tumor-promoting programs in BLCA. Selected MCPs are involved in neoplastic transformation and disease progression through partially overlapping yet molecularly distinct oncogenic programs affecting ECM remodeling, stromal activation, angiogenic reprogramming, and EMT/EMP. This section provides a compilation of the pro-tumorigenic properties of the best-supported MCPs in BLCA.

### 3.1. Immunodependent and Tumor-Intrinsic Signaling: OPN

Osteopontin (OPN), a member of the small integrin-binding ligand N-linked glycoprotein (SIBLING) family, is encoded by the *SPP1* gene [101]. Originally characterized as a bone matrix protein secreted by osteoblasts [102], it is now gaining attention as a multifunctional cytokine-like ECM protein in cancer [103]. OPN is overexpressed in tumor tissues, in different BLCA cell lines and patient plasma both transcriptionally and translationally [57,58,59,60], serving as a promising biomarker. Additionally, in MIBC, a gene expression comparison against healthy samples showed a tendency for upregulation of genes involved in the cell cycle and proliferation [61].

Initial studies did not find any correlation between OPN overexpression and cancer stage or grade [57]. However, it has become clear that OPN overexpression does correlate with T-stage, higher tumor grade, muscle invasiveness, and overall poorer patient outcome [57,58,59,60,61,62]. A mechanistic link of how OPN overexpression results in BLCA has not been completely confirmed, but some insights have been gathered from different functional approaches. Analyses of two BLCA datasets identified co-expression of two metastasis-associated genes (S100A8 and MMP9) upon OPN overexpression [58]; the authors then experimentally validated those results, showing that these genes are induced upon OPN expression [58]. In addition, in vitro OPN expression has been shown to increase invasive capacity through activation of the JAK1/STAT1 signaling pathway [59]; whether this pathway is also relevant in vivo remains to be tested. Molecular characterization of a possible mechanism of BLCA invasion has shown that OPN operates within the TME through a macrophage-dependent mechanism, where tumor-associated macrophage (TAM)-derived OPN binds to CD44 on cancer cells to activate the TIAM1–Rac1 pathway [63]. This signaling cascade is particularly important in early metastatic dissemination from BLCA tumors, as its inhibition in other cancers markedly suppresses lung metastasis while having limited effects on established lesions [63].

### 3.2. Proteolytic Remodeling and Metabolic Control: SPARC

SPARC (secreted protein acidic and rich in cysteine; osteonectin or BM-40), a calcium (Ca^2+^)-binding matricellular glycoprotein [104], illustrates a proteolytic and metabolic arm of this invasive network. Early clinical evidence in BLCA demonstrated that SPARC upregulation significantly correlated with histological grade, pathology state, invasiveness, as well as with poorer patient outcomes [83]. Notably, a significant positive correlation was observed between SPARC and MMP2 [83], a protein that breaks down ECM, implicating SPARC-driven proteolytic ECM remodeling as a plausible mechanism of a pro-invasive program. The observed clinical features were later supported by a proteomics study on a cell secretome from an aggressive BLCA cell line, in which both SPARC and MMP2 were detected, with SPARC being among the top significantly upregulated proteins in the secretome from highly metastatic cells in comparison with the less aggressive counterpart [84]. Functional assays focusing on SPARC neutralization, both in cell surface and conditioned media, showed cell motility reduction, positioning the protein as a key pro-migratory molecule. A mechanistic insight has not been elucidated as to how SPARC modulates cancer progression. However, given the upregulation of different proteases present in the secretome, it can be proposed that SPARC possibly modulates tumor–microenvironment interactions and ECM remodeling, thereby contributing to invasiveness [84]. Despite the mechanism of action of SPARC, its invasive program has been linked to metabolic regulation. The Aryl Hydrocarbon Receptor (AhR) was identified as an upstream transcriptional regulator of SPARC by chromatin immunoprecipitation profiling. Tryptophan metabolism driven by the Tryptophan-metabolizing enzyme (TDO2) activates AhR, which binds to the promoters of SPARC and FILIP1L (Filamin A Interacting Protein 1 Like), increasing their transcription and subsequently leading to tumor progression. Further clinicopathological follow-up confirmed that SPARC expression positively correlated with lymph node metastasis, advanced pathological stage, and overall poorer survival [85].

### 3.3. EMT Induction and Systemic Metastatic Niche Conditioning: Tenascins

The extracellular matrix glycoproteins of the tenascin family similarly intersect on MCPs-mediated invasive remodeling. Tenascin-C (TN-C), Tenascin-W (TN-W), Tenascin-X (TN-X), and Tenascin-R (TN-R) have been implicated in the progression of different types of cancer, yet the strongest evidence for pro-tumorigenic function currently exists only for TN-C and TN-W [105], with TN-C as the best studied in BLCA. Mechanistically, in BLCA, tumor cells, together with stromal mesenchymal cells, including fibroblasts, secrete TN-C. This molecule binds to the transmembrane proteoglycan syndecan-4 and activates the downstream NF-kB signaling pathway. This leads to the initiation of an EMT-like program: activation of the EMT-associated transcription factor Snail, an increase in the expression of the mesenchymal markers N-cadherin, vimentin, and ECM remodelers MMP2 and MMP9, while a decrease in the expression of the epithelial marker E-cadherin. Altogether, these changes enhance proliferation, migration, and invasion [64]. That study only used samples from Chinese patients, and therefore, it is important to cover more populations as this is an important variable in cancer progression [106].

TN-C contribution, however, is not limited to modulating the local TME but broadens to the conditioning of pre-metastatic niches at secondary sites. Histological examination of benign lymph nodes from patients affected by MIBC, who later developed metastases, demonstrated high levels of TN-C. Importantly, extracellular vesicles (EVs) in urine samples from MIBC patients containing cytokines such as TGF-β, HGF, bFGF, and KGF/FGF7 showed a positive correlation with lymph node TN-C expression. Furthermore, in vitro experiments confirmed that BLCA-derived EVs induce TN-C expression in primary fibroblasts through NF-κB (Nuclear factor kappa-light-chain-enhancer of activated B cells) signaling, collectively suggesting an EV-dependent mechanism of priming future metastatic sites in regional lymph nodes [65]. Caution must be taken as this study used a rather small sample size and one lymph node per patient, and more samples are needed to use it as both a marker and target in BLCA.

The role of TN-C seems to be type- and isoform-specific. TN-C variants containing specific domains become concentrated in newly formed vessels, especially in the stroma of papillary tumors [66], suggesting a possible role in stromal remodeling and angiogenesis, while another study found limited expression of TN-C in NMIBC [67]. Overall, the findings suggest a possible involvement of TN-C in local and systemic regulation of BLCA carcinogenesis that warrants further validation. Locally, it remodels the primary TME by inducing EMT and stromal activation, while systemically it conditions pre-metastatic niches through EV-mediated fibroblast reprogramming. This dual effect distinguishes TN-C as one of the few MCPs in BLCA with documented roles in both primary invasion and distant metastasis sites.

### 3.4. Stromal Reprogramming and Pro-Invasive Signaling: CCN1 and CCN2

Members of the CCN family can induce pro-invasive programs but are also involved in stromal reprogramming in BLCA, with CCN1 (also known as Cysteine-rich angiogenic inducer 61 (CYR61)) and CCN2 (also known as connective tissue growth factor (CTGF)) showing the most BLCA-relevant evidence. CCN1 is upregulated in both tumor samples and in the urine of BLCA patients and is associated with poor prognosis. Transcriptomic comparison of NMIBC versus MIBC patient samples identified CCN1 as one of the most significantly upregulated transcripts in the invasive state, a finding further confirmed in additional samples [69]. Gene silencing assays using different cancer cell lines confirmed that CCN1 promotes cell migration in BLCA and invasion in MIBC-derived cell lines. The mode of action was not determined, but CCN1 overexpression correlated with the upregulation of the TME and EMT remodelers MMP2 and NRP1 (Neuropilin-1) [69]. Additionally, an integrated bioinformatics analysis further linked CCN1 and TN-C to microenvironment-associated gene signatures associated with poor survival, supporting its relevance not only as a tumor-associated marker but also as part of a broader stromal-invasive program [70]. This concept is further reinforced by evidence from co-culture experiments of bladder mesenchymal stromal cell biology, where CCN1 has been shown to be not only a marker but also a downstream effector in the tumor–stroma platform. Exosomal miR-217 derived from bladder mesenchymal stromal cells activates the Hippo–YAP pathway in BLCA cell lines, subsequently upregulating YAP targets, including CCN1, thereby promoting proliferation, migration, and suppressing apoptosis in BLCA cells [71]. Similarly, CCN1 was linked to another representative of the CCN family, CCN2, via the same signaling axis in BLCA. Both proteins were identified as YAP-associated downstream effectors in patient samples, while the tumor suppressor gene *RASSF1A* showed an inverse relationship with their expression. To illustrate in vitro, loss of *RASSF1A* expression was correlated with increased CCN1 and CCN2 levels, while restoration of *RASSF1A* activated the Hippo signaling and reduced expression of both genes [72]. Notably, CCN2 itself supports oncogenic programs. Its expression positively correlates with BLCA aggressiveness and induces proliferation and migration of BLCA cells, with Akt and Erk signaling as plausible players in the process. Complementary in vivo evidence confirmed that CCN2 knockdown significantly reduces tumor growth in a xenograft mouse model. Targeting this protein made BLCA cells more sensitive to mitomycin, a compound used in chemotherapy, leading to cell apoptosis [73].

The latest studies also support the pro-tumorigenic role of CCN2. Zhang and colleagues (2025) [74] used a large sample size and confirmed significant downregulation of CCN2 transcript and protein in BLCA tissue. Notably, residual high CCN2 expression was associated with poorer overall survival (OS) and correlated with immune cell infiltration patterns, including shifts in macrophage and T-cell subsets [74]. The pro-metastatic role of stromal CCN2 has been further substantiated at the CAF subtype level. A pan-cancer single-cell transcriptomic analysis, including BLCA, identified syndecan 1 (SDC1)-expressing CAFs as a conserved, tumor-enriched fibroblast cell subtype whose abundance correlates with lymph node metastasis and poor OS in cancer patients. Within this subtype, CCN2 is the primary secreted effector—its knockdown in SDC1+ CAFs significantly reduced BLCA cell migration and invasion in vitro and lymph node metastasis in vivo. SDC1 CAF-derived CCN2 was found to activate FGFR3 signaling in tumor cells, inducing EMT, with the upstream KLF6–CTGF–FGFR3 axis identified as a complete pro-metastatic regulatory cascade [75]. Together, CCN1 and CCN2 are part of a feed-forward loop where their tumorigenic activity is amplified by stromal inputs (exosomal miR-217, loss of RASSF1A), sustaining proliferation, migration, and survival. This interaction positions the CCN family as coordinators of a tumor–microenvironment crosstalk rather than autonomous oncogenic drivers.

### 3.5. CAF-Mediated Remodeling and Paracrine EV Signaling: Periostin

Periostin (encoded by the gene *POSTN*) shares several functional characteristics with members of the CCN family. In the broader urothelial context, immunohistochemistry evaluation of upper urinary tract urothelial carcinoma samples revealed that high stromal POSTN expression was associated with hallmarks of aggressive local behavior, such as higher pathological tumor stage and invasion to lymph nodes and blood vessels, suggesting that periostin induces stromal programs associated with tumor invasiveness [95]; however, this analysis was performed using a small sample size. In BLCA, periostin is enriched in MIBC-derived EVs from patient samples and correlates with a worse prognosis [96]. Periostin-borne EVs can also be found in MIBC cell lines [96]. EVs with high periostin content enhance migration and invasion of low-grade BLCA cells in vitro by a paracrine effect, accompanied by ERK activation. RNA interference of *POSTN* decreases integrin β1, N-Ras, and phosphorylated ERK levels, resulting in reduced invasive capacity of cancer cells and changes in cell morphology, with fewer protrusions required for ECM degradation [96]. A recent single-cell transcriptomic study identified a population of periostin+ CAFs as pro-tumorigenic in BLCA. These cells were abundant in tumor tissues and exhibited transcription programs associated with angiogenesis, migration, invasion, and cell cycle regulation. The IL1B/IL1R1 axis, a pro-inflammatory cytokine-receptor pair, was found to mediate CAF-monocyte communication, contributing to an immunosuppressive TME. The authors validated the computational results using in vitro knockout models of periostin in CAF and co-culture experiments [97]. Additionally, interaction of periostin+ CAFs with endothelial cells could impact vascular remodeling and angiogenesis. Notably, this mirrors the TAM-dependent OPN secretion previously described [63], where MCP-driven invasion in BLCA is not a cell-intrinsic property of malignant cells but a combined force of different stromal cell populations such as macrophages, CAFs, and endothelial cells.

### 3.6. Microenvironment-Dependent Invasive Signaling: Decorin

Decorin (DCN) belongs to the SLRP family [107]. In vitro experiments using murine bladder tumor cell lines have found DCN to be a secreted protein promoting angiogenesis and invasion. Functional experiments revealed that its silencing decreased tumor growth and impaired invasion, while its overexpression had the opposite effects. The study later found that decorin overexpression also occurs in samples from patients with aggressive MIBC in comparison to NMIBC and a healthy bladder. A handful of genes linked to adhesion and migration programs were also co-expressed with DCN in this sample set. It is important to point out that, even though there was a distinct cellular immune profile between non-invasive and invasive disease, DCN was not among the main leaders driving this process [91]. Altogether, DCN appears to contribute to invasive programs, but its broader biological role remains highly context-dependent and can be shaped by the TME. Thus, more studies are needed to contextually consider DCN as a plastic MCP, with its pro-tumorigenic capacity in MIBC likely reflecting microenvironmental reprogramming.

### 3.7. Summary

Overall, tumor-promoting MCPs in BLCA belong to a network of interconnected pro-carcinogenic programs including, but not limited to, promotion of invasion and migration (OPN, SPARC, DCN), activation of stromal networks (CCN1 and CCN2), ECM remodeling and EMT (TN-C, periostin), and conditioning of pre-metastatic niches (TN-C, periostin). Rather than functioning as independent oncogenic drivers, these proteins act as context-dependent mediators that integrate signals across tumor, immune, and stromal compartments. We encourage the field to include larger sample sizes, functional experimentation, as well as in vivo validation in order to clarify correlation and causation. Only then will this perspective help reframe MCPs as components of a coordinated extracellular regulatory system and, importantly, suggest that the same regulatory architecture may be repurposed for tumor suppression under different biological conditions, as discussed in the following section.

## 4. Matricellular Proteins Involved in Tumor-Suppressing Programs

Cancer-promoting properties of MCPs are not evenly shared across all family members. A subset of MCPs has primarily tumor-suppressive signatures in BLCA with limited reported pro-tumorigenic roles. Others, such as periostin, SPARC, and DCN, can exhibit context-specific duality: the same protein functioning as either an oncogenic promoter or suppressor under specific biological conditions. These characteristics underscore the functional plasticity of MCPs, whose role is primarily determined by the microenvironment in which they operate. Anti-cancerogenic actions of these MCPs involve inhibition of angiogenesis and tumor growth, supporting epithelial integrity and suppression of invasion, as well as induction of apoptosis.

### 4.1. Anti-Angiogenic Signaling and Vascular Restraint: Thrombospondin-1 (TSP-1)

Among the primarily cancer-suppressing MCPs, thrombospondin-1 (TSP-1), encoded by the *THBS1* gene, represents one of the most extensively studied examples. TSP-1 is a matricellular glycoprotein best characterized as an endogenous inhibitor of angiogenesis [108,109,110], acting through interaction with CD36 and CD47 cell surface receptors, initiating apoptosis and suppressing vascular endothelial growth factor (VEGF)-dependent vessel sprouting [111,112,113,114]. This axis has been actively targeted as a therapeutic route through the development of VT1021, a TSP-1-stimulating small molecule in clinical evaluation [115,116]. In BLCA, reduced perivascular THBS1 expression by immunohistochemistry was identified as a progression factor from superficial to muscle-invasive disease [76], while low THBS1 expression independently correlated with higher tumor grade, disease recurrence, and adverse clinical outcome [77,78]. Importantly, THBS1 loss correlated with p53 alterations and increased tumor vascularity [77], suggesting inhibition of angiogenesis as a possible primary mechanism of TSP-1-mediated tumor suppression. At the genetic level, the THBS1-1223 A/G polymorphism in the Chinese population, which reduces transcriptional output, was correlated with a higher recurrence risk in BLCA patients [79], indicating that the presence of polymorphisms near the gene might make some populations more susceptible to this type of cancer. In addition, a sex-specific hormonal axis has been proposed: androgens suppress *THBS1* expression in a male mouse model of BLCA, and castration restored TSP-1 levels, reducing malignant growth [80], offering a plausible molecular mechanism and an overview of the benefits of anti-androgenic therapies to the biased male predominance of BLCA incidence. However, recent studies have not found significant associations between THBS1 expression and BLCA progression [89,117]. While more research is needed to directly link TSP-1 expression as a restrainer in BLCA, these studies lead the way to position TSP-1 as a versatile tumor suppressor integrating vascular, genetic, and hormonal axes. Its loss during the transition from non-invasive to invasive disease may shift the extracellular balance from an anti- to a pro-angiogenic state in BLCA.

### 4.2. ECM Stabilization and Epigenetic Silencing: Fibulins (FBLN1 and FBLN5)

A similar wide tumor-suppressive activity has been documented for members of the fibulin family of ECM glycoproteins across multiple cancer types [118,119,120]. Two family representatives, Fibulin-5 (FBLN5) and Fibulin-1 (FBLN1), also showed consistent suppressive evidence specifically in BLCA. Both were downregulated in BLCA samples compared to normal tissue [81,82], highlighting a common pattern of loss during tumorigenesis. FBLN1 downregulation was largely attributed to promoter hypermethylation, linking epigenetic silencing to disease progression and recurrence, particularly in NMIBC [81]; nonetheless, whether the epigenetic silencing of FBLN1 is a universal pattern in BLCA samples or is associated with a specific population remains to be determined. Restoration of either FBLN1 or FBLN5 expression suppressed key malignant phenotypes, including cell proliferation, migration, and invasion [81,82]. Furthermore, FBLN1 also inhibited angiogenesis and promoted apoptosis in BLCA in vitro models [81]. The epigenetic basis of FBLN1 loss is notable in the broader context of MCP suppression in BLCA. This raises the possibility that the erosion of ECM-stabilizing programs can precede drivers of genomic instability and may represent a permissive event in early BLCA evolution rather than a late consequence of disease aggression.

### 4.3. Cell Cycle Control, ROS Regulation, and Inflammatory Suppression: SPARC

The earliest evidence of SPARC function in BLCA came from gene mapping after allele deletion on chromosome 5, which led to bladder neoplasia [121]. Consistent with this genetic evidence, urothelial cells transformed by exposure to the environmental carcinogens cadmium and arsenite showed a near-complete loss of SPARC at both the mRNA and protein levels [86]. However, the study did not provide a whole-genome mutational assessment upon carcinogen exposure, and therefore, it is unknown whether SPARC loss was a primary or secondary response. Interestingly, a follow-up study demonstrated that although SPARC expression was restored by stable transfection to endogenous levels (or even higher), a population of tumor-initiating cells suppressed SPARC expression while retaining tumorigenicity following transplantation into immunocompromised mice [87], suggesting an intrinsic silencing mechanism. Using mouse-deficient SPARC mutants, it was later revealed that this protein directly suppresses tumor progression of BLCA via two different mechanisms. On the one hand, SPARC has an antiproliferative effect by arresting cancer cells at the G1/S cell cycle checkpoint with upregulation of p21 and p27, which act as inhibitors of cyclins A, D, and E. On the other hand, SPARC decreases the accumulation of reactive oxygen species (ROS) and the resulting DNA damage. Another important function of SPARC is the inhibition of a pro-inflammatory microenvironment. Co-cultured experiments of cancer and normal urothelial cells revealed that SPARC restricts a feed-forward loop by inhibiting the NF-kB and AP-1 pathway by downregulation of TGF-β and SDF1; this is usually a contribution from CAFs and TAM. Collectively, these events significantly decrease angiogenesis [88]. The contrast between tumor-suppressive and tumor-promoting SPARC activity described earlier is striking. It seems that the same protein is engaged in opposite programs depending on whether it is being silenced by carcinogens or transcriptionally activated by tryptophan metabolism. Overall, this implies that the functional identity of SPARC in BLCA is tightly linked to the regulatory context.

### 4.4. Growth Factor Sequestration and ECM Signaling Inhibition: Decorin

DCN demonstrates a well-reported history of anti-tumorigenic properties in a wide range of cancers [122] and its anti-tumorigenic properties are evident in both human bladder tissues (malignant and non-malignant) and BLCA cell lines, which lack expression of this protein [92]. Consistently, restoration of DCN expression in BLCA experimental models repressed cell proliferation, underscoring a tumor-suppressive function [92]. In BLCA, circulating concentration levels of DCN have been reported to be lower in serum of a small cohort of BLCA patients, in comparison to healthy controls [93]. The same tendency was found in the analysis of tumor samples, where both mRNA expression and protein levels of DCN were lower [93,94] or even absent [92] compared to normal samples. These anti-tumorigenic effects of DCN are mediated by two different mechanisms: first, cell cycle arrest at the G1/S is achieved by increasing the protein expression of p21, a cyclin-dependent kinase inhibitor. Secondly, DCN binds to TGF-β1, inhibiting the pathway, especially the activity of the downstream effector MMP2. Inhibition of the cell cycle promotes apoptosis, while reduced TGF-β1 ensures cell–cell adhesion and decreased migration [94]. DCN is also involved in cell invasion, which is achieved by negative regulation of IGF-1R signaling as decorin binds the receptor and modulates the stability of downstream effectors in the pathway [123]. Interestingly, DCN and SPARC share similar tumor-suppressive mechanisms in BLCA. They both enforce G1/S checkpoint control while simultaneously disrupting extracellular feedback loops that sustain malignant progression. On the other hand, their capacity to promote invasion in MIBC emphasizes that suppressive activity is a microenvironmentally conditioned state rather than an intrinsic property.

### 4.5. EMT Suppression and mTOR Inhibition: Periostin

Periostin also shows functional plasticity supporting or restraining bladder carcinogenesis. Kim and colleagues (2005) [124] reported a significant reduction in POSTN expression in BLCA tumors and cell lines, which correlated with a more advanced disease state. Follow-up experiments showed that ectopic expression of periostin suppressed the invasiveness of BLCA cells in vitro without altering cellular proliferation, suggesting that its suppressive activity is selectively directed at the invasive and metastatic cascade rather than at bulk tumor growth [124]. Mechanistically, periostin facilitated the downregulation of the EMT-associated transcription factor Twist and the transcriptional upregulation of E-cadherin, thereby reinforcing epithelial adhesion events [98]; validation is needed to confirm this also occurs in vivo. In addition, the mTOR pathway, a critical regulator of cell growth, invasion, and survival [125], was attenuated in periostin-expressing BLCA cells, as evidenced by reduced phosphorylation of PDK1, Akt, and the downstream mTORC1 target S6 ribosomal protein [99]. Thus, the suppressive function of periostin in BLCA seems to be compartment-specific as well. If expressed in tumor cells, it enforces epithelial restraint through EMT suppression and mTOR inhibition, while if present in CAFs, the same protein drives invasion, angiogenesis, and immune evasion.

### 4.6. Summary

**Figure 2:**
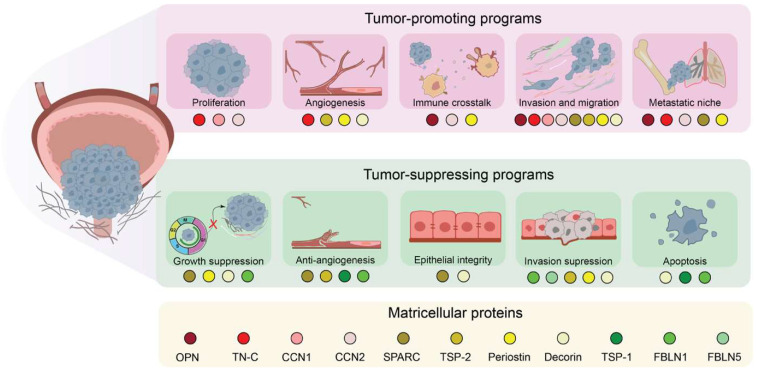
Schematic overview of the major biological programs regulated by selected MCPs in BLCA. MCPs participate in both tumor-promoting and tumor-restraining processes. Tumor-promoting programs include proliferation, invasion/migration, metastatic niche formation, angiogenesis, and immune crosstalk. Tumor-restraining programs include growth suppression, invasion suppression, apoptosis, anti-angiogenesis, and maintenance of epithelial integrity. Colored circles indicate MCPs implicated in each biological process based on current evidence in BLCA. Several MCPs exhibit functional duality and are involved in both programs. OPN, osteopontin; SPARC, secreted protein acidic and rich in cysteine; TN-C, tenascin-C; CCN, cellular communication network; connective tissue growth factor; TSP, thrombospondin; FBLN, fibulin.

Overall, tumor-suppressive MCPs in BLCA converge on several common biological processes, including inhibition of angiogenesis (TSP-1), ECM stabilization and epigenetic regulation (fibulins), induction of cell cycle arrest (SPARC and DCN), proliferative and invasive control (DCN), and suppression of epithelial plasticity (periostin) (Figure 2). Yet, this functional duality complicates understanding of the true biological functions of MCPs in BLCA, underscoring the need for deeper analysis of the factors driving this functional switch.

## 5. Putative Determinants of MCP-Associated Functional Variability

**Figure 3:**
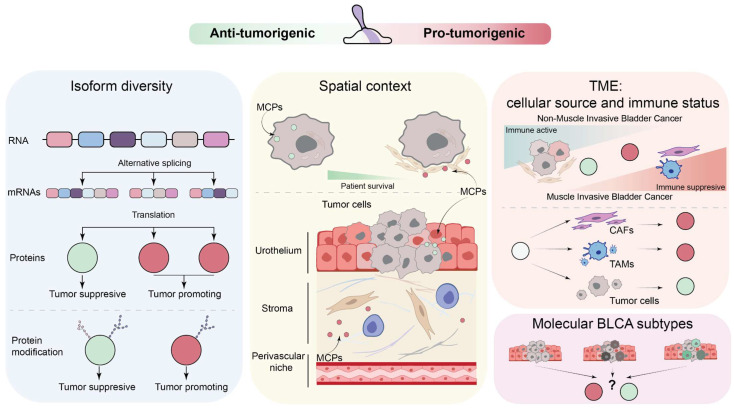
Putative context-dependent factors that may contribute to MCP-associated functional variability in BLCA. Isoform diversity, spatial compartmentalization, TME composition, and BLCA molecular subtypes are presented as candidate determinants that may contribute to variability in MCP-associated phenotypes in BLCA. The degree of support for each factor varies across individual MCPs, and in many cases, these relationships remain to be proven in BLCA-specific mechanistic studies. **Isoform diversity:** Alternative splicing can generate multiple protein isoforms from a single transcript. In selected MCPs, distinct isoforms may be associated with divergent functional effects, including tumor-promoting or tumor-restraining phenotypes (upper panel). Post-translational modifications, such as glycosylation, may further modify protein interactions and downstream signaling independently of splicing (lower panel). **Spatial context:** In addition to expression within cancer cells, MCP-associated effects may vary according to spatial localization within the tissue (upper and lower panels). Differential localization in tumor, stromal, or perivascular compartments may be associated with distinct biological and prognostic outcomes. **TME:** cellular source and immune status. As BLCA evolves, the TME may shift from a relatively immune-active state toward a more immunosuppressive and immune-evasive configuration. This evolving cellular landscape may influence which cell populations produce MCPs and how MCP-associated signals are interpreted within the tumor microenvironment. **Molecular BLCA subtypes:** Molecular subtypes differ in tumor cell differentiation and stromal/immune composition and may therefore influence MCP-associated phenotypes. Yet, their direct effects on MCP function remain largely unknown. Green and red circles represent MCPs reported to be associated with tumor-restraining and tumor-promoting phenotypes, respectively; these labels reflect context-specific findings and do not imply fixed functional identities. CAFs, cancer-associated fibroblasts; MCPs, matricellular proteins; TAMs, tumor-associated macrophages; TME, tumor microenvironment. “?” indicates the uncertainty of the molecular subtypes of BLCA on MCPs’ function.

Although the literature supports a context-dependent role for several MCPs in BLCA, the strength of evidence differs substantially across proteins, ranging from expression-based associations to direct functional and mechanistic studies. Consistent with this, multiple studies have reported biological variability or even contradictory findings regarding the roles of MCPs in BLCA. In some contexts, the same MCP is associated with both tumor-promoting and tumor-restraining programs. Although such variability may reflect genuine biological complexity, it may also be influenced by differences in study design, experimental models, and patient cohorts. MCP-associated effects are therefore unlikely to be explained by a single factor and should instead be interpreted within a broader contextual framework that includes both intrinsic molecular features and extrinsic microenvironmental cues. Understanding the role of a given MCP in BLCA thus requires moving beyond expression-level observations to consider the conditions under which the protein is produced, processed, localized, and interpreted by the surrounding tissue. In the following subsections, we discuss candidate contextual factors that may contribute to divergent MCP-associated functions in BLCA, while acknowledging that those associations have not been equally addressed across individual proteins and therefore, in many cases, they remain inferential rather than directly demonstrated (Figure 3).

### 5.1. Isoform Diversity

Isoform diversity, driven mainly by alternative splicing, is a fundamental mechanism that expands the proteomic repertoire of an organism; in the context of cancer, specific splicing isoforms can influence neoplastic transformation and progression differently [126,127]. Generation of a variety of different but related protein isoforms can affect functional domain compositions, post-translational modification sites, and protein–protein interactions [128,129,130]. Such changes may influence protein activity in ways that support or restrain carcinogenesis. Cancer cells often intervene with the splicing machinery to favor the expression of tumor-promoting protein forms [131,132,133]. Specific onco-isoforms can influence protein stability, subcellular localization of the protein, enzymatic activity, and enhance pathological signaling, thereby supporting aberrant proliferation and angiogenesis, escaping apoptosis, and enabling immune evasion [127,134,135,136,137]. Other isoforms may acquire or retain tumor-suppressive potential and promote apoptosis, inhibit proliferation, and the metastatic cascade [127,134,135,136,137]. Thus, the equilibrium between pro- and anti-cancerogenic protein forms could be an essential determinant in cancer biology.

In BLCA, several studies provide evidence of MCP isoforms that are dependent on functional divergence, with periostin being one of the clearest examples. As already discussed, growing evidence shows that periostin is implicated in both tumor promotion and suppression. At the same time, POSTN has been reported to have at least ten different isoforms in normal and malignant tissues [138]. This protein has several important domains: the cysteine-rich N-terminal domain (EMI domain), the Fasciclin I–like domain (FAS-1), and the C-terminal variable domain [139]. Early mutational analysis identified the C-terminal part of the protein as a hub of all alternative splicing events. Splicing of this region is sufficient to determine the functional activity of periostin, suggesting that isoform-specific differences within this region may underlie its opposing functional outputs across different contexts [124]. Later studies identified several periostin isoforms expressed in the bladder: the complete 23-exon wild-type mRNA, the isoform lacking exons 17, 18, and 21, the isoform lacking exons 17 and 21, and the isoform without exons 17 and 18. Among them, the canonical transcript was absent in bladder transitional cell carcinomas as well as in some cell lines, while the other isoforms were detected only in a subset of samples. Moreover, exclusion of exons 17, 18, and 21 results in more aggressive cancer phenotypes, supporting invasiveness and metastasis. The wild-type and the variant lacking exons 17 and 21 substantially reduce invasiveness and spread to the lung [100]. Additionally, it has been shown that the full-length C-terminus can bind up to 143 different proteins in vitro [140]. Different isoforms might only be able to bind to some of these proteins or bind a new subset of proteins, thereby altering downstream effects. Overall, these findings suggest that a specific isoform composition may help explain the dual role of periostin in BLCA.

Generally described as a tumor-suppressive molecule, thrombospondin-2 (TSP-2) extends the concept of isoform-dependent functional switching to the vascular compartment of BLCA. The anti-angiogenic properties of full-length TSP-2 are determined by its thrombospondin type 1 repeat domain, whose loss abolishes inhibition of VEGF-A-induced endothelial proliferation and migration. A short TSP-2 isoform lacking this domain has been found to be expressed at substantially higher levels than the full-length TSP-2 in blood vascular endothelial cells of invasive BLCA. Critically, this short isoform exerts a dominant-negative effect, inhibiting wild-type TSP-2 activity when co-expressed. Accordingly, while overexpression of full-length TSP-2 reduced tumor vascularization, overexpression of the short isoform substantially increased it [90]. This represents an example in BLCA of an endogenous angiogenesis inhibitor being converted into a pro-angiogenic molecule through alternative transcription in tumor stromal cells, illustrating that isoform switching can not only abolish tumor-suppressive function but actively invert it.

The pathological weight of the isoform imbalance is reflected in clinical data. The full-length TSP-2 expression was inversely associated with T stage, metastasis, and grade of BLCA, and its loss correlated with increased MMP-9 expression. Critically, the 4N1K peptide derived from the C-terminal cell-binding domain shared by TSP-1 and TSP-2, but predominantly associated with TSP-2 expression in BLCA tissue, emerged as an indicator of metastasis-free survival, an effect linked to anti-angiogenesis [89]. Together, these findings suggest that domain-level composition can be a determinant of TSP-2 functional output in BLCA, positioning the isoform equilibrium between the wild-type and short dominant-negative variant as a critical switch between vascular restraint and tumor progression.

Tenascin-C provides, perhaps, the strongest tissue-based support for the idea that isoform diversity is linked to invasive BLCA behavior. This protein contains three domains: EGF-like repeats, fibronectin type III-like repeats (FNIII), and a terminal fibrinogen-like globular domain. Importantly, 9 of the 17 types of FNIII domains can be alternatively spliced, resulting in isoforms ranging from a small compact domain to a large unspliced variant. Among them, several TNC variants have been implicated in BLCA [141]. While in homeostasis, the large TNC isoforms are almost absent, early histopathological works and expression analyses identified large unspliced and oncofetal TNC forms (A1, B, and/or D domain-containing isoforms) in urothelial bladder carcinoma (UBC). Specifically, they were associated with tumor progression, muscle invasiveness, and vascularization [66,67].

Alternative splicing events were also described for other MCPs, such as osteopontin [142,143,144,145,146], CCN2 [147] and CCN1 [148], affecting different types of cancer, yet the specific role of these isoforms in BLCA remains largely unexplored.

DCN broadens this concept beyond classical alternative splicing alone. A recent study discovered that DCN function can be determined by isoform composition. Consistent with early data, upregulation of DCN was identified in MIBC, yet the glycosylation-deficient but not the wild-type form was the main driver of this observation. The glycosylated form was described as more tumor-suppressive, while the non-glycosylated one exhibited pro-tumorigenic capacity. Specifically, overexpression of the isoform lacking glycosylated sites prompted cancer stemness in both in vivo and in vitro models, while the canonical form had the opposite effect and reduced stemness-associated markers (CD44, CD49f, and ALDH1A1), lowering the proportion of CD44+CD49f+ cells, and suppressing proliferation, migration, and self-renewal [149]. As a result, the findings highlight that the biological impact of MCPs depends not only on total transcript abundance but also on isoform composition, some being pro-tumorigenic while others are anti-tumorigenic.

### 5.2. Spatial Context

A conventional view of protein biochemistry stipulates that the functional identity of a protein is encoded in its sequence [150], domain architecture [151], and folding [152]. Yet, proteins do not act in isolation but are embedded within complex, dynamic environments where the spatial organization of a given protein can profoundly influence its behavior [153,154]. Depending on the subcellular localization and microenvironmental compartment it operates in, the same protein may be involved in different interactions and execute context-dependent functions [154,155].

The concept is particularly relevant for MCPs. Unlike structural ECM proteins that are relatively fixed in their matrix context, MCPs are secreted and capable of operating across multiple compartments simultaneously [19,45,52]. As a result, the same MCP can be present in the tumor epithelium, the reactive stroma, and the perivascular niche at the same time, participating in different molecular pathways. In BLCA, the TME is spatially organized into biochemically distinct zones, which have a unique set of cells and signaling networks [10,56]. Therefore, spatial variability could be considered as a determinant of MCP functional output.

The prognostic value of TN-C in BLCA seems to be different depending on the expression pattern. Diffuse stromal accumulation of TN-C was associated with advanced disease and poorer survival. In contrast, cytoplasmic TN-C expression in tumor cells (particularly within the invasive front) has been linked to more favorable outcomes and independently predicts improved OS [68]. Notably, these opposing prognostic effects of the same MCP within a single tumor might reflect distinct biological programs, emphasizing spatial compartmentalization as the critical determinant over total expression levels.

Alteration in the spatial compartmentalization of SPARC was also observed during BLCA. In normal bladder tissue, SPARC is expressed across all urothelial layers and in sub-urothelial stromal cells [86,88,156]. Similarly to normal tissue, in NMIBC SPARC is retained in both the tumor epithelium and adjacent stroma. With invasive progression, SPARC demonstrates a spatial shift towards predominance in the stromal compartment with a marked reduction in uroepithelial cells [88]. Paradoxically, another study found that higher SPARC transcript levels correlated with more advanced disease [83]. This apparent contradiction could be explained when the spatial compartmentalization of a protein is considered. The clinical significance of SPARC in BLCA seems to depend not on the amount of SPARC, but on its location. In advanced disease, increased SPARC levels might mainly come from the surrounding stroma, while tumor cells themselves reduce or lose SPARC expression. This could explain why higher total SPARC can still be linked to worse outcomes. Consistent with this interpretation, in human BLCA microarrays, tumor cell-associated SPARC, but not stromal SPARC, correlated with disease-specific survival [88].

Collectively, these findings highlight the importance of considering the spatial origin of a protein, rather than relying only on the magnitude of expression. Spatial compartmentalization, therefore, could be a potential determinant of MCP function in carcinogenesis and a critical requirement for accurate mechanistic and prognostic interpretation.

### 5.3. Tumor Microenvironment

The TME can be another key determinant of MCPs’ functional switching, acting at the level of the tissue ecosystem. Its importance becomes evident when comparing the expression patterns and behaviors of MCPs in the normal bladder with those in evolving BLCA. In homeostasis, the normal bladder is characterized by continuous immune surveillance by resident immune cells and epithelial defense mechanisms [157], an undisturbed basement membrane [158], and generally low MCP expression in adult tissues [18]. In a stable state, MCPs are usually involved in ECM regulation, preservation of tissue integrity, and controlling cell proliferation [18,48,52]. During the development and evolution of BLCA, the microenvironment undergoes dramatic changes. These include changes in immune response, transformation of stromal composition, and reorganization of ECM [159,160,161,162]. As BLCA progresses from NMIBC to MIBC, relatively immune-active TME shifts towards a more immunosuppressive and immune-evasive state, although both stages exhibit substantial heterogeneity [163,164,165]. Therefore, the evolving milieu potentially alters both which cells produce MCPs and which receptors and cofactors are available to interpret their signals, collectively affecting functional output.

An immunosuppressive environment can act as a permissive gate for the pro-invasive program. To illustrate, immunosuppressive cell populations secrete MCPs with pro-carcinogenic properties, such as periostin derived from CAFs [97], CCN2 secreted by CAFs [75], and OPN secreted by TAMs [63]. These proteins are then involved in tumor progression by enhancing invasion, angiogenesis, and immune evasion, as reviewed in previous sections. Notably, the functional effects of MCPs are context-dependent: the same protein can exert opposing roles depending on its cellular source, as exemplified by periostin, which has been reported to suppress invasive capacity when expressed by BLCA cells [99]. Therefore, these observations suggest that the microenvironmental context acts as a determinant of MCP function, rather than MCP expression alone.

In this context, two important issues deserve further consideration. First, most available data are correlative, and the causal relationship between MCPs and the TME remains poorly defined. It is still unclear whether microenvironmental changes induce expression of MCPs or whether MCPs themselves reshape the TME in ways that favor tumor progression. Second, the prevailing view that MCPs mainly promote tumor growth overlooks their documented tumor-suppressive functions. Overall, these findings underscore the reciprocal relationship between MCPs and the TME and the need for a more mechanistic understanding of this interaction in BLCA.

### 5.4. Molecular BLCA Subtypes

An additional contextual dimension that may contribute to MCP-associated variability in BLCA is the molecular subtype. The classification frameworks indicate that BLCA is a rather heterogeneous disease composed of transcriptionally and microenvironmentally distinct tumor states [166]. In particular, MIBC has been classified into consensus transcriptional classes including luminal papillary, luminal non-specified, luminal unstable, stroma-rich, basal/squamous, and neuroendocrine-like tumors [167,168].

These subtypes broadly reflect differences in tumor cell differentiation state together with variation in stromal and immune composition. The luminal classes are characterized by related but distinct epithelial transcriptional programs, whereas the basal/squamous subtype reflects a different differentiation state; the neuroendocrine-like class represents a rarer transcriptional program, and the stroma-rich subtype is distinguished by a prominent contribution of non-malignant stromal signals [167]. Because these classes differ in biological organization and microenvironmental composition, they could provide a useful framework for interpreting MCP-associated phenotypes in a more context-resolved manner.

This dimension might be particularly relevant in the case of MCPs, whose biological effects are closely linked to extracellular signaling, fibroblast activity, vascular remodeling, and immune–stromal communication [17,18,50]. It is therefore plausible that the same MCP may exhibit distinct associations or functional outputs depending on whether it is expressed in a luminal, basal/squamous, stroma-rich, or neuroendocrine-like setting. In particular, tumors with prominent stromal signals may provide conditions in which MCP abundance reflects fibroblast-rich and ECM-remodeled microenvironments more strongly than tumor cell-intrinsic programs alone.

At present, however, direct BLCA-specific mechanistic evidence linking individual MCPs to defined molecular subtypes remains limited. For this reason, molecular subtype should presently be regarded as a plausible contextual framework for interpreting divergent MCP-associated findings rather than as an established determinant of MCP function across the entire protein family. This consideration may be especially important when evaluating proteins such as SPARC [83,88,156], periostin [98,99,124], tenascin-C [64,65,66], and CCN2 [73,74,75], whose reported functions are closely intertwined with stromal composition, ECM remodeling, and tumor–microenvironment crosstalk.

Molecular subtype may also help explain at least some of the discrepancies reported across the BLCA MCP literature. Studies based on bulk tumor material often do not distinguish whether MCP-associated signals arise predominantly from malignant cells or from stromal and immune compartments, and differences in subtype composition between patient cohorts may therefore influence both expression patterns and clinical associations. Incorporating subtype-aware analyses into future studies may thus improve interpretation of MCP biology and help clarify under which biological conditions individual MCPs contribute to tumor-promoting or tumor-restraining programs. However, molecular subtyping remains an evolving framework, with variable assignment across platforms and intratumoral regions, and current evidence is still insufficient to support routine use in clinical decision-making.

## 6. Implications for Immunotherapy Responses

Current BLCA treatment strategies operate through mechanistically distinct but convergent routes. The standard therapy for high-risk NMIBC, Bacillus Calmette-Guérin (BCG), delivers a live, attenuated strain of *Mycobacterium bovis* directly into the bladder, inducing immune-mediated cytotoxicity and an innate-to-adaptive immune cascade: bacterial attachment, immune cell recruitment, and tumor cell death, including apoptosis, necrosis, pyroptosis, and immune-mediated killing, that ultimately depends on a functional, T-cell-permissive TME [169]. For BCG-unresponsive disease, immune checkpoint inhibitors block the PD-1/PD-L1 interaction that tumors use to inactivate cytotoxic T cells, while antibody-drug conjugates deliver cytotoxic payloads directly to tumor cells via a surface marker; the two are increasingly combined, since conjugate-induced cell death can recruit T cells into poorly infiltrated tumors, improving overall survival [170]. Notably, the efficacy of each approach depends not only on tumor-intrinsic antigenicity but also on stromal and immune cell accessibility—an axis governed in part by the MCPs discussed above, with CAF-driven ECM remodeling and TGF-β signaling already recognized as major drivers of immune-excluded, treatment-resistant BLCA phenotypes [170].

Periostin is one of the MCPs that exemplifies this dependency: while tumor cell-derived periostin suppresses invasiveness by restraining EMT and inhibiting mTOR, periostin secreted by CAFs recruits M2-polarized macrophages via an IL1B/IL1R1 axis, generating an immunosuppressive stroma that plausibly limits how well BCG and immune checkpoint blockade can engage T cells within the tumor, regardless of the tumor’s baseline immunogenicity [97].

A parallel paradox emerges with TSP-1: although its loss during the NMIBC-to-MIBC transition removes a key anti-angiogenic restraint, TSP-1 can independently bind CD47, a receptor T cells normally use to sense their surroundings and regulate their own activity, on tumor-infiltrating CD8+ T cells, pushing them into a dysfunctional, exhausted state that limits their ability to attack the tumor. This means that restoring TSP-1 to counter angiogenesis could, in principle, simultaneously undermine the T-cell reinvigoration that immune checkpoint blockade is designed to achieve [171].

TN-C adds a further layer of complexity. Its role in conditioning pre-metastatic lymph node niches via EVs, and its ability to drive an EMT program in BLCA by binding syndecan-4 and activating the NF-κB signaling pathway, overlap mechanistically with its documented capacity to trap CD8+ T cells within the stroma. This works by binding the chemokine CXCL12, preventing them from reaching and attacking tumor cells—a phenomenon recognized as a confounder of checkpoint blockade efficacy across solid tumors [172,173].

Taken together, these findings suggest that MCP-driven changes to the TME and stroma may help explain why patients respond so differently, and sometimes unexpectedly, to BCG, immune checkpoint blockade, and antibody-drug conjugates in BLCA. Yet, as with the promoting and suppressing programs described earlier, direct causal evidence linking specific MCP isoforms or compartments to treatment outcome in BLCA patients remains limited, and dedicated correlative studies within clinical trial cohorts are needed to move this framework from mechanistic plausibility to clinical relevance.

## 7. Concluding Remarks and Outlook

MCPs are nonstructural ECM-associated regulators that have been associated with both tumor-promoting and tumor-restraining phenotypes in BLCA. The evidence is strongest for context-dependent behavior of selected proteins, including periostin, SPARC, TSP-2, and decorin, whereas for other MCPs the proposed functional variability remains based largely on expression patterns, prognostic associations, or evidence from non-BLCA systems. Isoform diversity, post-translational modification, cellular source, spatial compartmentalization, tumor microenvironmental composition, and molecular subtype provide plausible frameworks for interpreting divergent MCP-associated findings. Yet, these factors should not yet be considered universal or independently proven determinants of MCP function in BLCA.

A better understanding of the flexibility of MCPs requires careful consideration of several constraints. Routine use of pan-antibodies and pan-oligonucleotides targeting conserved regions fails to distinguish between isoforms [174,175,176]. This can lead to misinterpretation of the results, as expression levels consist of a mix of functionally distinct forms. Differences among experimental models may also contribute to apparently conflicting MCP-associated findings. The small number of BLCA cell lines further limits the ability to define the true role of MCPs. Cellular models do not fully represent the biological complexity and the heterogeneity of human tumors [177,178]. These include variation in the genetic and molecular characteristics of BLCA cell lines, the presence or absence of stromal and immune components, and differences between conventional two-dimensional culture systems, three-dimensional models, and co-culture systems. As mentioned above, simplified experimental systems may not fully recapitulate the spatial, cellular, and molecular complexity of the human BLCA microenvironment and should therefore be considered when interpreting divergent findings.

Comparisons across studies are additionally complicated by patient heterogeneity [179,180,181]. This includes inconsistent staging criteria [182] and methodological variability in handling tissue samples [183]. Common reliance on bulk transcriptomics does not provide compartmental resolution [184] and does not consider context-dependent dynamics. Using isoform-specific oligonucleotides and antibodies, single-cell resolution methods, consideration of the spatial distribution of the proteins, and standardization of the methodological procedures and participant pool may help produce more accurate and mechanistically informative insights into MCPs biology. Moreover, future studies could combine isoform-aware perturbation experiments with spatial, single-cell, and clinically annotated datasets to determine when individual MCPs exert tumor-promoting or tumor-restraining effects.

From a translational perspective, the context-dependent behavior reported for selected MCPs may provide opportunities for biomarker development and therapeutic investigation. Future strategies should account for MCP isoform composition, cellular source, spatial distribution, and microenvironmental context, where technically feasible. However, clinical translation will require robust validation of MCP-associated phenotypes in well-annotated cohorts and demonstration that targeting a specific MCP or MCP-associated pathway improves therapeutic response in BLCA. Thus, MCPs should currently be regarded as context-sensitive candidate regulators rather than uniformly actionable therapeutic targets.

Overall, MCPs likely operate as context-dependent regulators of BLCA rather than fixed tumor-promoting or tumor-suppressing factors. The multifaceted identity of MCPs is unlikely to be a contradiction and could be acknowledged as a core principle of their biology. Deciphering the contextual system that governs this switch holds great potential for interpreting their behavior and facilitating the development of next-generation effective treatments for BLCA.

## Figures and Tables

**Table 1:** Functional roles of matricellular proteins in bladder cancer.

MCP	Gene	Family	Pro(+)/Anti(−) Tumorigenic	Model	Short Rationale	Reference
OPN	*SPP1*	SIBLING	+	Clinical cohort; human tissues	Associated with poor prognosis and aggressive clinicopathologic features; promotes proliferation and invasion.	[57]
				Reanalyses of BLCA patient datasets; BLCA cell lines	Higher expression was linked to advanced stage, higher grade, and poorer survival; downstream targets were identified.	[58]
				Human tissues; cell lines	Promoted proliferation, invasion, and JAK1/STAT1 activation; associated with high stage and poor prognosis.	[59]
				Clinical cohort; plasma samples	Higher plasma OPN was associated with higher stage, higher grade, and poorer survival in muscle-invasive bladder urothelial carcinoma.	[60]
				Clinical cohort; human tissues;	OPN was markedly overexpressed in invasive BLCA and associated with aggressive disease features.	[61]
				Clinical cohort; plasma samples	Preoperative plasma OPN correlated with muscle invasion and higher pathologic stage.	[62]
				Clinical cohorts; human tissues; mouse metastasis models; cell lines	Macrophage-derived OPN promoted BLCA invasion, clonal growth, and metastasis through CD44s/TIAM1/Rac1, and higher OPN correlated with aggressive disease and worse outcome.	[63]
TN-C	*TNC*	Tenascin	+	Clinical cohort; human tissues; cell lines	TN-C increased with tumor grade and promoted bladder cancer migration, invasion, proliferation, and EMT via syndecan-4/NF-κB signaling.	[64]
				Clinical cohort; human tissues; primary fibroblasts; cell lines	TN-C identified a pre-metastatic lymph node niche in MIBC and was induced by BLCA EVs in fibroblasts through NF-κB.	[65]
				Clinical cohort; human tissues	Tenascin-C splice variants were more strongly expressed in invasive BLCA, especially A1 and D domains, and were associated with higher stage and grade.	[66]
				Human tissues; cell lines; xenograft mouse model	Tenascin-C splice variants were differentially incorporated into tumor vessels and vessel walls, with perivascular Tn-C in renal cell carcinoma shown to be tumor cell-derived.	[67]
				Clinical cohort; human tissues	Diffuse stromal TN-C predicted worse overall survival, while cytoplasmic TN-C in tumor cells predicted better overall survival; invasive-cell TN-C was independently prognostic.	[68]
CYR61 (CCN1)	*CYR61*	CCN	+	Clinical cohort; tissues; urine; cell lines	CYR61 was higher in MIBC, predicted poorer survival, and promoted migration/invasion in invasive BLCA cell lines.	[69]
				Reanalyses of BLCA patient datasets	CYR61 was one of the TME-related prognostic genes in BLCA and contributed to a high-risk signature linked to poor survival.	[70]
				Cell lines	hBSC-derived exosomal miR-217 increased CYR61 via YAP signaling and promoted BLCA cell proliferation and migration.	[71]
				Clinical cohort; tissues; cell lines	CYR61 was upregulated in BLCA, rose with disease severity, and was a YAP target suppressed by RASSF1A/Hippo signaling.	[72]
CTGF (CCN2)	*CTGF*	CCN	+	Clinical cohort; tissues; cell lines	CTGF was a YAP target elevated BLCA and linked to disease severity; RASSF1A activation reduced CTGF and increased chemosensitivity.	[72]
				Clinical cohort; human tissues; cell lines; xenograft	Overexpressed in BLCA and promoted proliferation, invasion, and mitomycin C resistance.	[73]
				Reanalyses of BLCA patient datasets; TCGA; tissue	CTGF was downregulated in BLCA overall, but higher expression was associated with poor prognosis, immune-infiltration shifts, and therapy-response differences.	[74]
				Human tissues; primary CAFs; BLCA cell lines; co-cultures	SDC1+ CAF-derived CTGF promoted EMT, invasion, and metastasis through FGFR3 signaling.	[75]
TSP-1	*THBS1*	Thrombospondin	−	Clinical cohort	Reduced perivascular TSP-1 at presentation predicted progression to invasive disease.	[76]
				Clinical cohort	Low TSP-1 was associated with recurrence, poorer survival, higher microvessel density, and p53 alterations in invasive BLCA.	[77]
				Clinical cohort	Low TSP-1 was associated with recurrence, poor survival, higher microvessel density, and p53 alterations.	[78]
				Clinical cohort	TSP-1 -1223 A/G polymorphism was linked to shorter time-to-recurrence, and the GG genotype had the lowest TSP-1 mRNA expression.	[79]
				Transgenic mouse model; mouse tissue samples; BLCA cell lines	Androgens suppressed TSP-1; castration increased TSP-1 and reduced tumor growth, supporting an anti-angiogenic role.	[80]
Fibulin-1 (FBLN1)	*FBLN1*	Fibulin	−	Clinical cohort; BLCA cell lines and mouse model	Fibulin-1 was downregulated by promoter hypermethylation, and low expression predicted recurrence; restoring it reduced proliferation, invasion, angiogenesis, and tumor growth.	[81]
Fibulin-5 (FBLN5)	*FBLN5*	Fibulin	−	Clinical tissue cohort; cell lines	FBLN5 was downregulated in BLCA; ectopic expression suppressed proliferation and invasion in 5637 cells.	[82]
SPARC	*SPARC*	SPARC	+	Clinical cohort	High SPARC expression was associated with higher grade, invasive stage, worse survival, and higher MMP-2 expression.	[83]
				Cell line	SPARC was associated with the aggressive phenotype, and anti-SPARC antibodies decreased cell motility.	[84]
				Bioinformatics analysis; BLCA cell lines; mouse models	TDO2/AhR signaling increased SPARC, and SPARC tracked with malignancy and adverse prognosis in BLCA	[85]
			−	Cell lines; human specimens; mouse work	SPARC was repressed in cadmium/arsenite-transformed urothelial cells and absent in malignant tumor cells, suggesting loss of a suppressive/adhesion-regulating function.	[86]
				BLCA cell line; mouse model;	SPARC was suppressed in tumor-initiating urospheres, and the paper argues these cells have an intrinsic mechanism to silence SPARC; this supports a loss-of-SPARC, anti-tumorigenic pattern in this model.	[87]
				Xenograft and metastasis mouse models; BLCA cell lines; primary mouse cell lines; human tissue samples	SPARC loss accelerated bladder carcinogenesis and metastasis, while SPARC expression correlated with better survival and reduced inflammation, proliferation, and lung colonization.	[88]
TSP-2	*THBS2*	Thrombospondin	−	Human tissues	Higher TSP-2 was linked to lower stage, less metastasis, lower grade, reduced proliferation, lower MMP-9, and longer metastasis-free survival.	[89]
			+	Human tissue samples; BLCA cell lines; xenograft mouse model	A shorter TSP2 transcript in tumor vessels lacked the anti-angiogenic domain and lost TSP2’s inhibitory effects on endothelial proliferation, migration, tumor growth, and angiogenesis.	[90]
Decorin (DCN)	*DCN*	SLRP	+	Human tissue samples; cell lines; orthotopic and subcutaneous mouse models	Decorin was overexpressed in invasive BLCA, promoted angiogenesis and invasiveness, and its knockdown reduced tumor growth.	[91]
			−	Human tissue samples; BLCA cell lines; mouse model	Decorin was absent from malignant BLCA cells in vivo and in vitro, and restoring decorin reduced proliferation, supporting an anti-tumorigenic role.	[92]
				Clinical cohort	Decorin was reduced in patient serum and tumor tissue, and the authors proposed it as a potential diagnostic marker with an antitumor-associated pattern.	[93]
				BLCA cell lines	Decorin was lower in tumor tissue, and adding decorin inhibited T24 proliferation and metastasis while increasing p21.	[94]
Periostin	*POSTN*	Periostin	+	Human tissue	High stromal periostin expression was associated with adverse pathological features and independently predicted worse overall and cancer-specific survival	[95]
				BLCA cell lines and tissue	EV-borne periostin promoted aggressiveness and invasiveness in BLCA, and higher urinary/tissue periostin was associated with muscle-invasive disease and poor outcome	[96]
				Cell lines and reanalyses of BLCA patient datasets	POSTN+ CAFs were enriched in BLCA, linked to poor prognosis, and POSTN knockdown reduced CAF-driven T24 migration and invasion	[97]
			−	BLCA cell lines	Periostin upregulated E-cadherin, suppressed invasiveness, and decreased Akt phosphorylation, supporting an antitumor role in this context.	[98]
				BLCA cell lines; mouse model	Periostin suppressed invasion and orthotopic tumor aggressiveness, with reduced PDK1/Akt/mTOR signaling and no effect on proliferation.	[99]
				Human tissue; BLCA cell lines	WT periostin was lost in BLCA, while Variant I lost suppressive activity; WT and Variant II suppressed invasion/metastasis, supporting periostin as a tumor suppressor in bladder carcinogenesis.	[100]

## Data Availability

No new data were created or analyzed in this study. Data sharing is not applicable to this article.

## Abbreviations

AhR	Aryl hydrocarbon receptor
BCG	Bacillus Calmette-Guérin
BMP	Bone morphogenetic proteins
BLCA	Bladder cancer
CAFs	Cancer-associated fibroblasts
CCN	Cellular communication network
CIS	Carcinoma in situ
CTGF	Connective tissue growth factor
CYR61	Cysteine-rich angiogenic inducer 61
DCN	Decorin
ECM	Extracellular matrix
EGF	Epidermal growth factor
EMT	Epithelial–mesenchymal transition
EMP	epithelial–mesenchymal plasticity
EVs	Extracellular vesicles
FAP	Fibroblast activation protein-α
FBLN1	Fibulin-1
FBLN5	Fibulin-5
FILIP1L	Filamin A interacting protein 1 like
FNIII	Fibronectin type III
MCPs	Matricellular proteins
MIBC	Muscle-invasive bladder cancer
MMP	Matrix metalloproteinase
NF-κB	Nuclear factor kappa-light-chain-enhancer of activated B cells
NMIBC	Non-muscle-invasive bladder cancer
NRP1	Neuropilin-1
OPN	Osteopontin
OS	Overall survival
ROS	Reactive oxygen species
SDC1	syndecan 1
SIBLING	Small integrin-binding ligand N-linked glycoprotein
SLRP	Small leucine-rich proteoglycan
SPARC	Secreted protein acidic and rich in cysteine
TAMs	Tumor-associated macrophages
TDO2	Tryptophan-metabolizing enzyme
TGF-β1	Transforming growth factor β1
TME	Tumor microenvironment
TN-C	Tenascin-C
TN-R	Tenascin-R
TN-W	Tenascin-W
TN-X	Tenascin-X
TSP-1	Thrombospondin-1
TSP-2	Thrombospondin-2
UBC	Urothelial bladder carcinoma
VEGF	Vascular endothelial growth factor
